# The implementation of the bioequivalence certification policy in Chile: An analysis of market authorization data

**DOI:** 10.1371/journal.pone.0217334

**Published:** 2019-05-29

**Authors:** Warren A. Kaplan, Jorge Cárdenas, Cristián Mansilla, Tatiana Tobar, Veronika J. Wirtz

**Affiliations:** 1 Department of Global Health, Boston University School of Public Health, Boston, United States of America; 2 Ministry of Health, Chile, Santiago, Chile; NPMS-HHC CIC / LSH&TM, UNITED KINGDOM

## Abstract

**Background:**

Affordability is a key barrier to access to medicines. Generic medicines policies can address this barrier and promote access. Successful uptake of generic medicines depends, in part, on ensuring that these products are interchangeable with reference products. Typically, bioequivalence certification is established in order to demonstrate such interchangeability.

**Objective:**

To study the implementation of the bioequivalence certification policy in Chile.

**Methods:**

We used Chilean Market Regulatory Authority data for analysis to study the number of products that obtained bioequivalence certification, the time until bioequivalence certification and associated factors to obtain bioequivalence.

**Results:**

As of January 2017, out of 2,336 products with a valid market authorization containing at least one of the 167 APIs that required BE certification, 1,026 products actually have BE certification (1,026/2,336, 43.9% compliance). Where data were available, the time between submission of the market authorization as a bioequivalent product to final authorization by the national medicine regulatory authority for most products varied between 4–6 months. The fraction of all BE products containing a given API out of the total marketed products containing that API varies considerably, e.g. for the API olmesartan there was only a single BE product marketed, the API diclofenac had none.

**Conclusions:**

Although the implementation of Chile’s bioequivalence policy increased the number of bioequivalent products, over 50% of generic products requiring bioequivalence that did not obtain this certification. Also for some of the API none or very few BE products are marketed which limits the success of a substitution policy. Further studies are required to identify the apparent lack of incentives to obtain bioequivalence certification. Studies of sales volumes and prices of the products are needed to identify whether generic products without bioequivalence certification either become bioequivalent or eventually exit the market.

## Introduction

Price is one of the major barriers to access to medicines. [1] An increasing number of countries have implemented generic medicines policies to promote medicines access as they move towards Universal Health Coverage, in large part because generic medicines are usually less expensive than the innovator medicines [2]. According to the World Health Organization (WHO), generic medicines are a “pharmaceutical product, usually intended to be interchangeable with the innovator product, which is usually manufactured without a licence from the innovator company and marketed after the expiry of patent or other exclusivity rights.”[3] It is called an “unbranded generic” when it is sold under (international) non-proprietary name (INN). If sold under a brand name, it is a “branded generic”. An “innovator product” is “generally the pharmaceutical product which was first authorized for marketing (normally as a patented product) on the basis of documentation of efficacy, safety and quality according to requirements at the time of the authorization.” [3]

Two medicines are ‘bioequivalent’ as defined by the WHO, if they contain the same active ingredient(s) in the same dosage form, strength and route of administration and their bioavailabilities, “… in terms of peak (Cmax and Tmax) and total exposure (area under the curve (AUC)) after administration of the same molar dose under the same conditions, are similar to such a degree that their effects can be expected to be essentially the same.” [4] Typically, bioequivalence (BE) is established in order to demonstrate so-called “therapeutic equivalence” between generic, off-patent versions of a corresponding reference medicine such that the BE medicine products can be substituted with the full expectation that the generic medicine will produce the same therapeutic effect and safety profile as the originally indicated (comparator or reference) product. Put another way, the establishment of BE allows for the bridging of preclinical and clinical data associated with the reference product to the generic product [5]. Two products are therapeutically equivalent if they are pharmaceutically equivalent or pharmaceutical alternatives and, after administration in the same molar dose, their efficacy and safety effects are essentially the same when administered to patients by the same route under the conditions specified in the labelling. This can be demonstrated by appropriate equivalence studies, such as pharmacokinetic, pharmacodynamic, clinical or in vitro studies.” [3] In practical terms, the concept of “bioequivalence” can give legitimacy to generic medicines by implying that one commodity can be replaced by another and establishing the parameters for a market transaction based on price [5].

The requirements to obtain market authorization of a generic pharmaceutical product depends on the regulatory framework applied by the Medicines Regulatory Authority (MRA) of each country. In the United States and in the European Union (EU), apart from containing the same active ingredient, dosage and the like, proof of therapeutic equivalence with a comparator product is a pre-requisite for the market authorization of a generic product [6–7]. A comparator product (or referent) is defined as “a pharmaceutical product with which the generic product is intended to be interchangeable in clinical practice. The comparator product will normally be the innovator product for which efficacy, safety and quality have been established.” [3]

Many MRAs in Latin America do not require bioequivalence tests but merely proof of pharmaceutical equivalence. The World Health Organization considers products pharmaceutically equivalent if they contain the same molar amount of the same active pharmaceutical ingredients (APIs) “… in the same dosage form, if they meet comparable standards and if they are intended to be administered by the same route.” Two pharmaceutically equivalent products do not necessarily imply that they are therapeutic equivalents, as differences in the API solid-state properties, the excipients and/or the manufacturing process and other variables can lead to differences in product performance. [3] Therefore, some countries in Latin America (Argentina, Brazil, Mexico) distinguish those products with proof of therapeutic equivalence tests as true “generics” and those with pharmaceutical equivalence as “copy or similar medicines” [8].

The evaluation of bioequivalence data often presents a major technical and/or financial challenge for the companies and regulatory authorities. To the extent there are any local manufacturers, not all of them can carry out bioequivalence studies since these studies may require (1) access to the comparator product; (2) the capacity to carry out studies in healthy humans that compare the proposed generic product with the comparator product, including; (3) measurement of plasma concentrations of substances using a reliable, sensitive and specific assay [9].

The financial burden and effort required to perform BE analyses has led to the development of the ‘biowaiver’ process in many regulatory authorities. The term biowaiver is applied to a regulatory approval process when the dossier (application) is approved based on evidence of equivalence other than through *in vivo* bioequivalence testing. The WHO has proposed that many medicines on the Essential Medicines List (EML) can now be considered for a biowaiver procedure, eliminating the need for conducting in *vivo* bioequivalence testing for all products simultaneously [4]. In 2006, the WHO Expert Committee on Specifications for Pharmaceutical Preparations provided recommendations for bioequivalence testing of essential medicines [10].

### Experiences of Latin American countries introducing bioequivalent requirements

There has been a lack of thorough description and preliminary evaluation of the actual implementation and impact of bioequivalence policies in many countries in Latin America, with the possible exceptions of Mexico [11] and Brazil [12–15]. The situation regarding the requirements to demonstrate BE of marketed medicines has an uneven development in Latin America. Nonetheless, most countries have created “priority” lists of medicines to be taken under their BE requirement. Generally, prioritization has been based on the health risk and/or in some property of the molecule and/or on the epidemiology [5].

There is still wide variation across countries in the BE requirements [5]. In 1999, the Brazilian medicines regulatory authority (ANVISA, acronym in Portuguese) was established. In the same year, the “Generic Medicines Act” introduced the concept of bioequivalence into the Brazilian legal framework. Further, this law established a deadline requiring pre-approval of bioequivalence protocols by the regulatory authority as well as set the rules for conducting *in vivo* studies. It is also possible to waive *in vivo* BE studies (biowaiver). ANVISA created a fast track approval process for local firms prepared to register generic products and local industry adapted rapidly to this regulatory environment. [14].

Further, Brazil restricts generic substitution to BE products allowed in lists of authorized competing medicines, prescribed by their INN names and with distinctive labels [1]. In this regard, “generic substitution” by dispensers (e.g., pharmacies) allows them to replace an innovator product with an equivalent generic product, whether marketed under a trade name or generic name containing the same active ingredient(s). If doctors do not agree with a generic substitution, they must indicate, “substitution not allowed” on the prescription [13]. While doctors in Brazil’s National Health System are obligated to prescribe using the generic name, private physicians are not bound by this rule, and thus can continue to prescribe by brand name [13].

Argentina, also in 1999, published its first technical recommendations regarding the type of *in vivo* studies required to demonstrate BE and identified a priority list of APIs with a detailed schedule for BE testing for those products containing these APIs [5]. In Colombia, BE studies are required for immunosuppressants and anticonvulsants, and in 2009, the government required completion and submission of BE studies for all products containing small molecule APIs based on the “… most accepted international recommendations.” Bioequivalence studies are required for various therapeutic groups (e.g., anti-cancer, immunosuppressives, antiretrovirals and other groups) when sold in Colombia under generic or brand name, when the manufacturer requests a certification of interchangeability with the innovator in the market [16].

In Mexico, from 2010 all registered medicines (except innovator products) need to demonstrate therapeutic equivalence [15–17].

Chile started to introduce BE requirements in 2005 with its first legally binding resolution; i.e., “Rule which defines the criteria for establishing TE [therapeutic equivalence] in pharmaceutical products in Chile” (Exempt Resolution 727) [18–19]. Over time, more decrees were published and these incorporated more APIs. Finished products containing these API needed to demonstrate BE in order to be sold. For instance, Decree 500 established a list of 43 APIs (See S1 Table). Finished products containing these API had to demonstrate BE by December 2014. Decree 981/12 initiated program of gradual BE compliance for 49 additional APIs. Decree 864 of October 24th 2012 included 12 additional APIs.

Several technical guidelines, not shown in the S1 Table, were initiated in 2007, one of which is directed to *in vitro* ‘biowaiver’ requirements, although all the other technical guidelines required *in vivo* testing.

The Chilean Ministry of Health is responsible for publication of these decrees. Medicines included in the list of WHO prequalified medicines were recognized as BE by the Institute of Public Health of Chile (ISP acronym in Spanish) in 2009. Biowaiver studies or BE clinical trials are conducted by clinical research organizations and licensed laboratories in Chile. These studies are paid by the manufacturer. The manufacturer will submit the results of these studies to the national medicine regulatory authority in Chile to request approval of the product as bioequivalent in order to receive a BE certificate.

### Objective of the paper

The objective of this study is an analysis of the implementation of Chile’s bioequivalence policy in terms of number and type of BE products, the actual number of BE products approved for marketing, as well as factors associated with the speed of BE approval. We provide policy recommendations for Chile that may also be useful in other countries.

## Methods

### Data sources

We used the following three data sources:

1. Market authorization of pharmaceutical products
  For this study we used data from the Chilean Medicine Regulatory Authority on every product with an approved and valid (“vigente”) market registration as per January 2017. The data included the origin of the particular presentation, e.g., made in Chile or imported in various forms (e.g. bulk).
2. List of bioequivalent pharmaceutical products and their registrations
  We requested information about the officially registered BE pharmaceutical products containing a given active pharmaceutical ingredient from the Chilean Market Regulatory Authority. This information was as of 01/31/2017 and included the BE registration numbers, the dates of the official Decree that listed a given API as requiring bioequivalency when marketed as part of a finished product, the date that a BE application for a given pharmaceutical product containing a given API was submitted to the national medicine regulatory authority, the date that a particular product containing a given API became certified as BE, and the primary indication for the product containing that API. We excluded all sustained release, injectable and oral solutions from the analyses. We calculated the time needed for a given product containing an API to obtain BE certification by comparing the date of submission of a BE application and the official BE certification date. We also stratified the time needed to obtain BE certification by whether the APIs were considered under a ‘biowaiver’ or not.
3. Decrees of bioequivalents
  S1 Table lists 20 Decrees. These decrees specify the APIs for which a finished product containing any of these APIs requires BE certification and the final deadline dates for any product containing an API listed in a Decree had to receive a BE registration. In total 167 different APIs are listed in the Decrees.

### Data analysis

From the total number of products with valid market authorization, we extracted the non-sustained release, oral dosage forms. From those, we excluded all products which do not include any of the 167 APIs included in a Decree requiring BE. The final sample of oral, non-sustained-release and marketed products with approved (‘vigente’) market authorization were matched with a BE registration number reported in the database. For those BE compliant presentations that matched (i.e. that were approved for marketing), we also determined how many of these approved products with BE certification were made in Chile or imported into Chile. We analyzed the number of products that received BE certification as well as the total number of unique APIs that had at least one product that was certified as BE. We further studied the number of BE products per therapeutic group and the length of time (months) from submission of BE test results to certification for those BE registered products with market approval which had both starting and ending dates available. Approval times are relevant to ensure access to BE products without delay.

We used the non-parametric Spearman rank-order correlation coefficient to measure the strength and direction of association between a number of variables: (a) total number of products with a given API listed in any of the decrees regarding BE and (b) the percentage of total products containing that API that received BE certification (“compliance”), (c) percentage of marketed products with bioequivalent APIs and number of months to get BE approval, and (d) BE compliance for APIs using in vitro BE pathway and time to BE certification. The Spearman test was chosen because the variables do not need to be normally distributed and the correlation is not very sensitive to outliers. We used a two-tailed statistical significance of p < .05 to test the association. We calculated a two-sample Kolmogorov-Smirnov statistic to test whether the time distributions were the same for locally produced or imported products (i.e., both datasets are coming from the same distribution). We used Stata v. 8 [20] for correlation testing and Microsoft Excel for other analyses.

## Results

**Fig 1:**
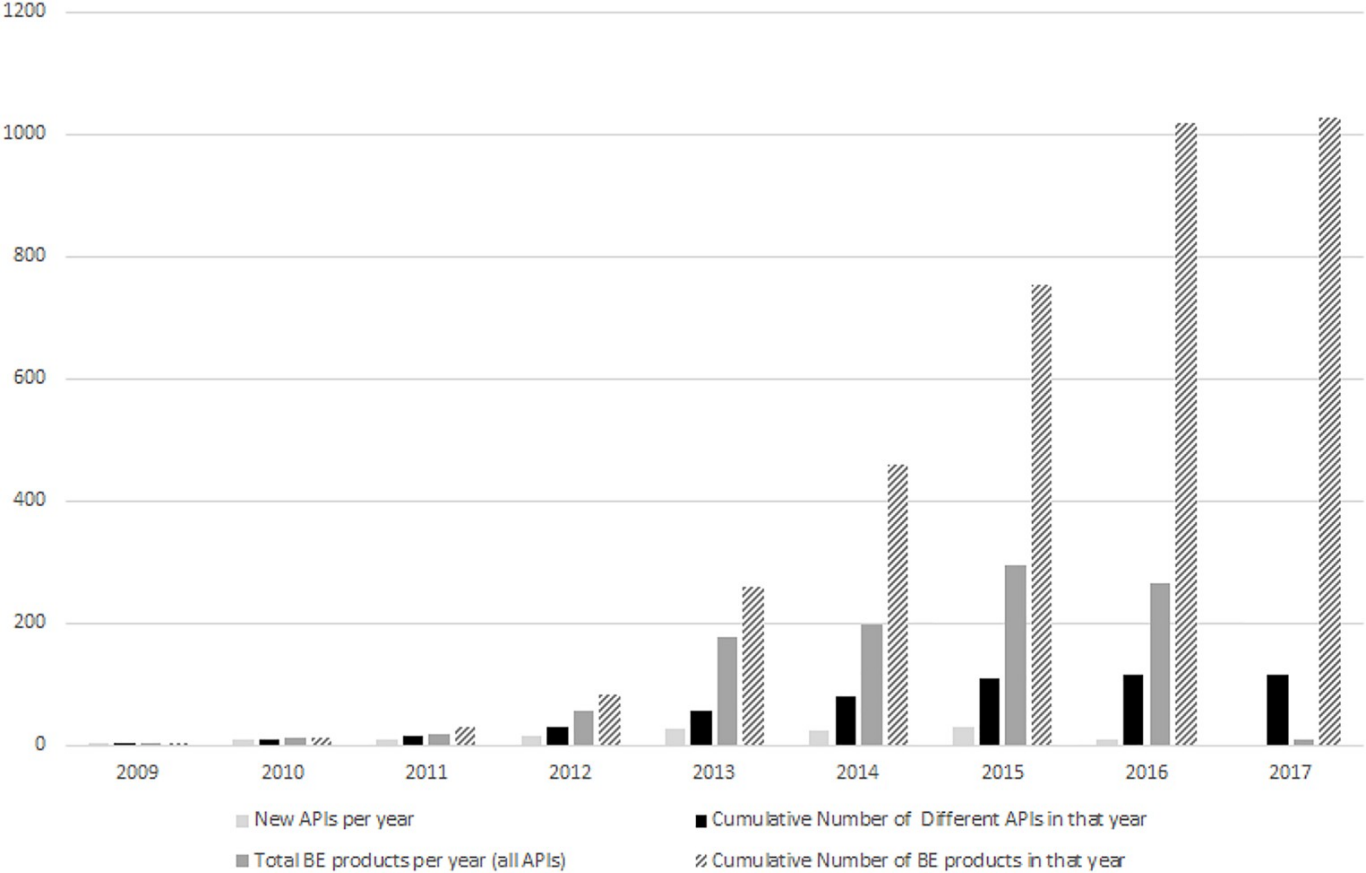
Yearly count of different APIs, cumulative APIs and total BE products.

Fig 1 below shows the number of different APIs deemed BE in a given year starting in March 2009 until 2017. The other bars show the cumulative total of all APIs in that year as well as the total BE products in that particular year for all APIs. There were 2,336 products with a valid market authorization containing at least one of the 167 APIs that required BE certification. Of those products, 1,026 products have BE certification (1,026/2,336, 43.9%, compliance).

**Fig 2:**
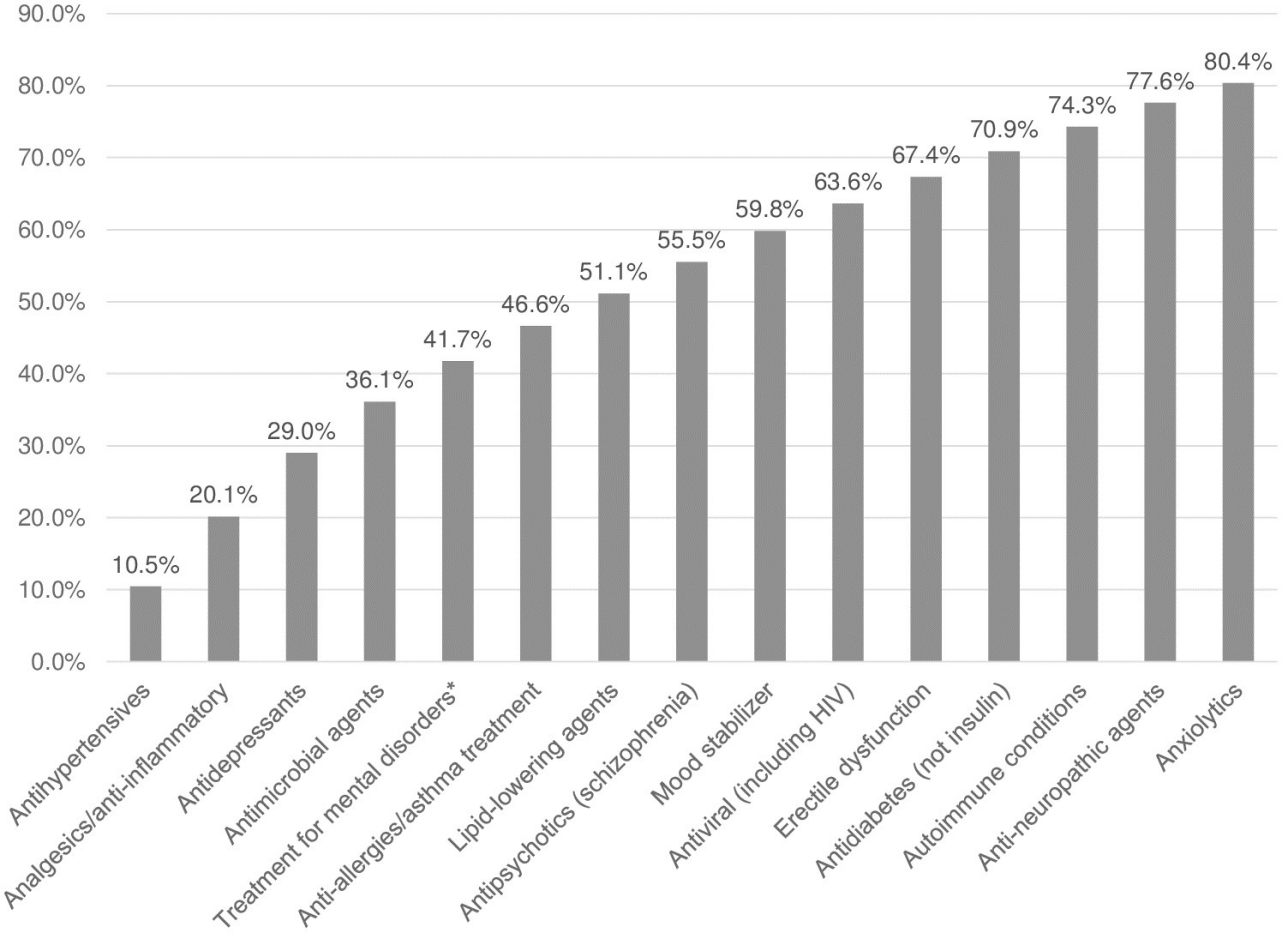
Cumulative percentage of bioequivalent products by therapeutic group. Slightly over 25% of the 1,026 products are intended for treatment of various mental and mood disorders.

Fig 2 shows the conditions that might be treated by these 1,026 BE products as of January 2017. Fifteen condition**s** or uses comprise 80% of all these BE products.

Out of the 2,336 products with a valid market authorization containing at least one of the 167 APIs that legally required BE certification, 48.6% were made in Chile, 17.8% were imported as finished product, and 26.6% were imported as finished and locally re-packaged. The remaining seven percent of products were imported as bulk or defined as “semifinished”.

**Fig 3:**
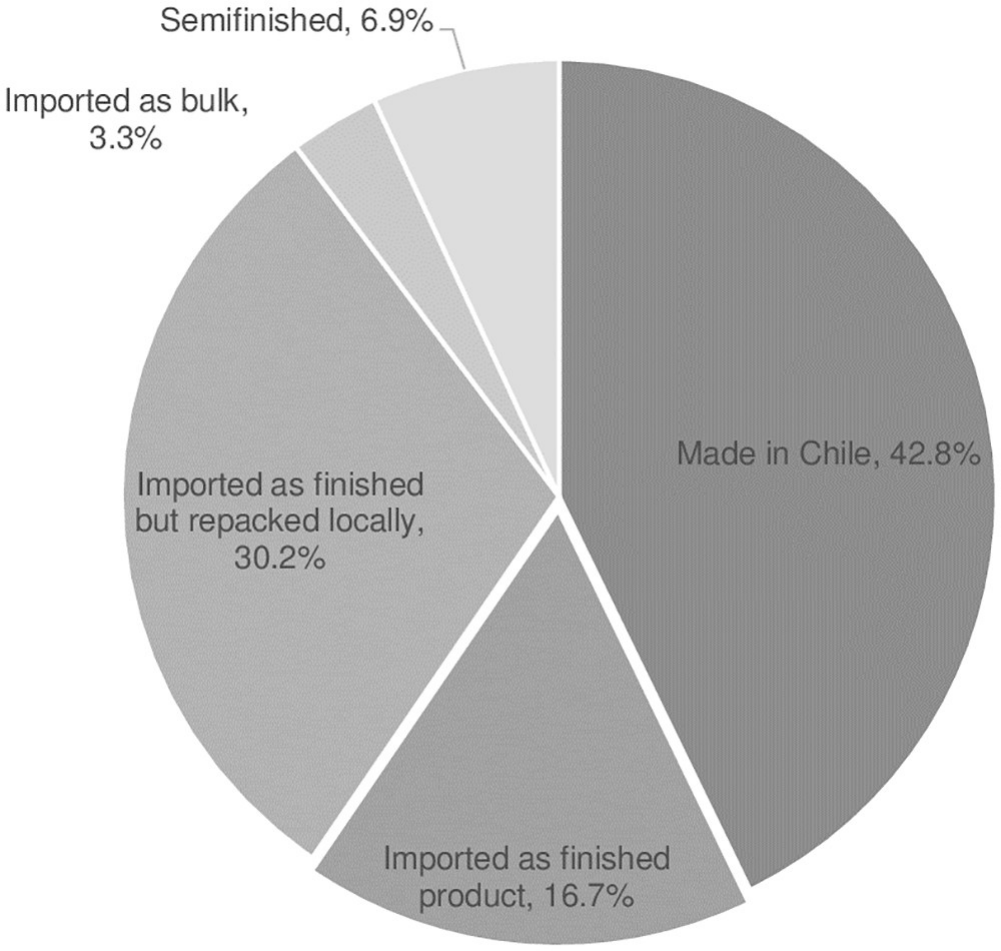
Percentage of BE products made locally or imported.

Of the 1,026 products marketed products that actually have received BE certification, about 43% are made locally, the rest being imported either as finished product or some other form—primarily in bulk form (e.g. tablets) and packaged and labelled in Chile (Fig 3)

**Fig 4:**
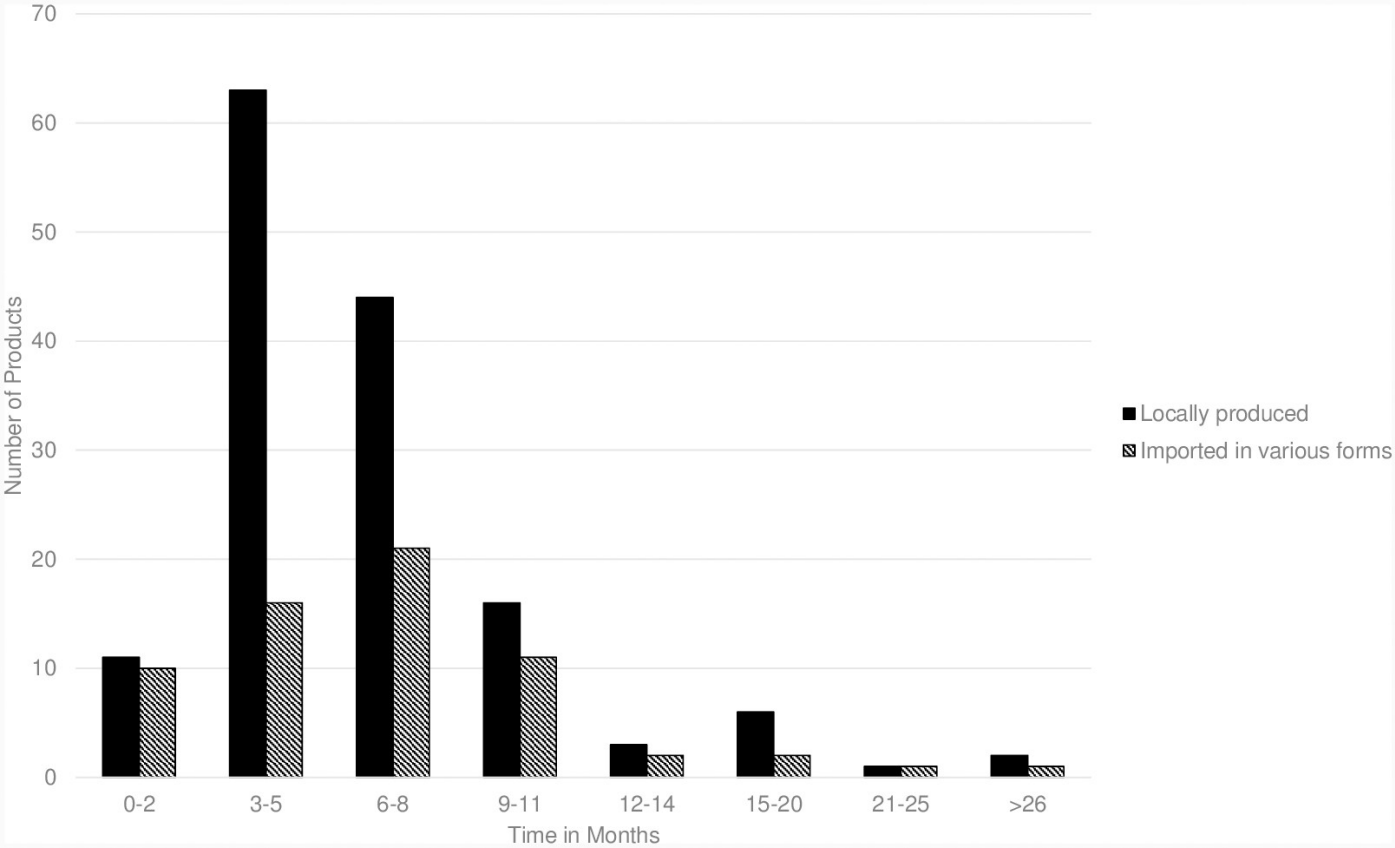
Distribution of BE products by length of time (months) to final BE authorization.

Fig 4 is the distribution of BE products where we were able to identify the time (months) from BE application date to final BE authorization.

The two-sample Kolmogorov-Smirnov statistic to test whether the time distributions were the same for locally produced or imported products (i.e., both datasets are coming from the same distribution) could not reject this test (p = 0.980). The time to approval does not vary much (mostly between 4–6 months) regardless of whether the API containing product is locally produced or imported.

The fraction of API-containing BE products out of the total marketed products containing that API varies considerably. As of January 1, 2017, olmesartan has a single validated product on the market but it is BE (= 100%). Diclofenac has 34 non-injectable, non-sustained release presentations on the Chilean market and none are BE. For a given API, the relationship between the total number of API-containing products on the Chilean market and the percentage of BE compliance for that API is weakly and insignificantly negative; Spearman’s coefficient = -0.074; p = 0.43.

The relationship between percentage of marketed products with bioequivalent APIs and number of months to get BE approval is also negative with a Spearman’s coefficient of -0.1138; (p = 0.1059) so the inference is very weak that faster approval times are associated with higher BE compliance. Some APIs of marketed products included in the study were allowed to present *in vitro* BE data under ‘biowaiver’: doxycycline, clomipramine, levonorgestrel, levothyroxine and chlorphenamine. For these 5 APIs (total n = 8) using an *in vitro* BE pathway, there is no association between faster approval time in months and greater compliance (Spearman’s coefficient = 0.04; p = 0.91).

## Discussion

This study of the implementation of the Chilean bioequivalence policy provides insight into the extent of uptake of bioequivalent products and the factors related to their uptake. We note the number of new APIs added by decree increased over time as did the cumulative number of registered BE products. Many important therapeutic groups such as analgesics and cardiovascular medicines had a high number of products with BE certification, potentially providing patients with lower priced products compared to the innovator products.

Overall, 43% of the 2,336 products that contained an API which legally required BE actually obtained it. We suggest that some continuing studies are needed to review the sales volumes and prices of the 57% of non-BE, products on the Chilean market with a view toward seeing if all non-BE products either become BE or eventually exited the market Possible explanations delays in obtaining the required BE certification may be the capacity of certified laboratories to do the testing. Given the complicated environment dictating BE policy, the quantitative factors influencing the percentage of BE compliance will likely be difficult to specify.

We found no relationship between the number of products on the market with a specific API that required BE certification and the percentage of these products with an actual BE certification One might surmise that APIs for which only a few products are marketed should get the most attention from regulators and thus should have the greatest BE compliance but this appears not to be the case. More investigations are warranted regarding BE compliance. It appears that both locally produced and imported medicines overall have about the same BE approval times, although our sample was limited to just 210 products for which we had the required information. Those biowaived medicines are primarily locally produced.

We have identified some factors that may hamper BE implementation. For glibenclamide, furosemide, cloxacillin, metoclopramide, chlorphenamine and ciprofloxacin, the reference product was never sold and it is certainly possible that a Chilean reference existed and was used as the BE comparator. Alternatively, the company making the comparator might have submitted a BE dossier but did not market the product. If a reference product never existed in Chile, then there would be a lack of data about the original product. More transparency is needed in policy documents about the rationale for selection of APIs. Clarification is also needed in cases in which the comparator API was different from the listed API requiring BE certification, as it was the case with isosorbide mononitrate listed as the referent for isosorbide dinitrate. [21]

The impact of policies designed to incentivize use of generic medicines has been studied in various countries [22–25]. First, doctors in Chile tend to prescribe pharmaceuticals of a specific brand, which cannot be legally substituted at the point of sale [26] and this, in principle, will impede the effect of BE policies. However, although not legally mandated, substitution of the specific brand (although not necessarily by a BE counterpart) prescribed by the doctor at the point of sale appears to be a common occurrence [26]. Although this may help consumers to gain access to less expensive pharmaceuticals, this is not always the case as pharmacy personnel are also subject to economic incentives by manufacturers for dispensing a higher number of units of particular brands [26]. Nonetheless, the fact that BE requirements now exist in Chile can significantly strengthen the policy position that physicians should prescribe pharmaceuticals by their INN, not their brand name [27]. This broadens consumer choice since users can replace prescribed pharmaceuticals with bioequivalent products without the need to acquire a new prescription from their doctor.

Important barriers to generic substitution of medicines also include information asymmetries related to the patient’s knowledge about the substitution policy itself and/or the existence of APIs that are BE in the Chilean market. Consumers may not know for which innovators have BE counterpart products available. Patients have to ask at the pharmacy if their medicine has a BE version rather than presented by default with the BE version instead of the innovator.

The study has several important limitations. Our analysis was limited to the time period 2009 to 2016. We are depending upon the accuracy of information from the Chilean regulatory authorities which may be subject to some errors. The study focuses on market registration data and not the actual sales and/or prices of the products. Even though sales data are important and should be studied in the future, analyzing the market registration of BE products is a first and necessary step to fully understand the impact of the BE policy on the Chile market. There are also a very limited number of ‘biowaived’ products so in our view statistical comparisons with non-biowaived products are not warranted.

## Conclusions

Implementation of Chile’s BE policy increased number of BE products although there is still some distance to go to attain BE for all products and remove non-BE products from the market. Also for some of the API none or very few BE products are marketed which limits the success of a substitution policy. From an administrative viewpoint, continued monitoring and transparency in the BE registration process would be important. It is encouraging that important therapeutic groups contain products that are now BE.

## Supporting information

S1 Fig (XLSX)

S2 Fig (XLSX)

S3 Fig (XLSX)

S4 Fig (XLSX)

S1 Table Table of BE decrees. (DOCX)

## References

[1] Gonzalez CPV, Fitzgerald J, Rovira J. Generics in Latin America: Trends and regulation. J. Generic Medicines. 2008; 6:43–56.

[2] Cameron A, Ewen M, Ross-Degnan D, Ball D, Laing R. Medicine prices, availability, and affordability in 36 developing and middle-income countries: a secondary analysis. Lancet 2009; 373: 240–249. 10.1016/S0140-6736(08)61762-6 19042012

[3] World Health Organization. Quality Assurance of Medicines Terminology Database—List of Terms and related guideline. https://www.who.int/medicines/services/expertcommittees/pharmprep/20171109ReportQASTerminology_database.pdf?ua=1

[4] World Health Organization. Proposal to waive in vivo bioequivalence requirements for WHO Model List of Essential Medicines immediate -release, solid oral dosage forms. 2006; Annex 8, WHO Technical Report Series No. 937. Geneva, World Health Organization.

[5] Fonseca EM. Relevance of variation in use of terminology to define generic pharmaceutical products. Rev. Panam. Salud Publica. 2015; 37: 113–117. 25915016

[6] US Food and Drug Administration. Approved products with Therapeutic Equivalence (Orange Book) 2018; 38^th^ Edition, Washington DC https://www.fda.gov/ucm/groups/fdagov-public/@fdagov-drugs-gen/documents/document/ucm071436.pdf.

[7] European Medicines Agency. Guideline on the investigation of Bioequivalence. 2010. Doc. Ref.: CPMP/EWP/QWP/1401/98 Rev. 1/ Corr 2010. http://www.ema.europa.eu/docs/en_GB/document_library/Scientific_guideline/2010/01/WC500070039.pdf.

[8] Homedes N, Ugalde A. Multisource drug policies in Latin America: survey of 10 countries. Bull. World Health Organization. 2005; 83:64–70. PMC2623457 15682251

[9] Hill S, Johnson K. Emerging challenges and opportunities in drug registration and regulation in developing countrieis. DFID Health Systems Resource Centre 2004. http://www.heart-resources.org/wp-content/uploads/2012/10/Emerging-challenges-and-opportunities-in-Drug-registration-and-regulation.pdf.

[10] WHO Expert Committee on Specifications for Pharmaceutical Preparations. Report 40. Annex 7. Technical Report No. 937. Geneva, World Health Organization. 12768889

[11] Moïse P, Docteur E. Pharmaceutical Pricing and Reimbursement Policies in Mexico. OECD Health Working Papers. 2007; 62 pp. https://www.oecd.org/mexico/38097348.pdf.

[12] Gomes Barra AC, de Albuquerque I. A decade of generic pharmaceutical policies in Brazil J. Generic Medicines. 2011; 2:72–75.

[13] Fonseca EM, Shadlen KC. Promoting and regulating generic medicines: Brazil in comparative perspective. Rev. Panam. Salud Pública. 2016; 41:1–6. 10.26633/RPSP.2017.5 PMC6612747 28444005

[14] Fonseca E. Reforming pharmaceutical regulation: a case study of generic medicine policy in Brazil. Policy Soc. 2014;33:65–76.

[15] Shadlen K, Fonseca E. Health policy as industrial policy: Brazil in comparative perspective. Politics Soc. 2013;41:560–586.

[16] Storpirtis S, Gai MN, Cristofoletti R. Generic and similar products in Latin American countries: Current aspects and perspectives on bioequivalence and biowaivers. Pharmaceuticals, Policy and Law. 2014; 16:22–248.

[17] González PE, Barraza Lloréns M, Working for the health of the population: proposal of a pharmaceutical sector policy. [In Spanish: Trabajando por la salud de la población: Propuestas de política para el sector farmacéutico. Versión para el diálogo.] 2011. Ciudad de México: Funsalud.

[18] Fiscalia Nacional Economica, Santiago Chile. Study on the effects of bioequivalence and the penetration of generics in the field of free competition [In Spanish: Estudio sobre los effectos de la bioequivalencia y la penetration de genericos en el ambito de la libre competencia] September 2013. http://www.fne.gob.cl/wp-content/uploads/2013/09/estu_001_2013.pdf.

[19] Comparative study of prices of Products: Bioequivalent v. Reference, Metropolitan Area, March 2013 [In Spanish: Estudio comparativo de precios de Productos: Bioequivalentes v/s de Referencia, Área Metropolitana, March 2013, Santiago Chile. https://www.sernac.cl/wp-content/uploads/2013/05/informe_bioequivalentes_marzo2013.pdf.

[20] Stata StataCorp LLC, College Station TX. https://www.stata.com/

[21] Mansilla Cristián, Cárdenas Jorge, Kaplan Warren A., Wirtz Veronika J, Kuhn-Barrientos Lucy, de Zárate Matías Ortiz, Tobar Tatiana, Herrera Cristian A. Evaluation of the effects of a generic substitution policy implemented in Chile. BMJ Global Health, 10.1136/bmjgh-2018-000922 30899555 PMC6407566

[22] Kaplan WA, Ritz LS, Vitello M, Wirtz VJ. Policies to promote use of generic medicines in low and middle income countries: a review of published literature, 2000–2010. Health Policy. 2012;106:211–224. 10.1016/j.healthpol.2012.04.015 22694970

[23] Moye-Holz D, van Dijk JP, Reijneveld SA, Hogerzeil HV. Policy approaches to improve availability and affordability of medicines in Mexico—an example of a middle income country. Global Health. 2017; 13:53 10.1186/s12992-017-0281-1 28764738 PMC5540413

[24] Dylst P, Vulto A, Simoens S. Demand-side policies to encourage the use of generic medicines: an overview. Expert Rev. Pharmacoecon. Outcomes Res. 2013; 13:59–72. 10.1586/erp.12.83 23402447

[25] Vogler S. The impact of pharmaceutical pricing and reimbursement policies on generics uptake: implementation of policy options on generics in 29 European countries-an overview. Generics and Biosimilars Initiative Journal. 2012; 1:93–100. 10.5639/gabij.2012.0102.020

[26] Sanitary Code of Chile, 11 December 1967, Article 101: codified 14 February 2014. https://www.leychile.cl/Navegar?idNorma=5595.

[27] Organisation for Economic Co-operation and Development. Competition Issues in the distribution of pharmaceuticals- Contribution from Chile. Global Forum on Competition. 2014; Document DAF/COMP/GF/WD(2014)3. http://www.fne.gob.cl/wp-content/uploads/2017/10/oecd_01_2014.pdf

