# Supplementary material for: The implementation of the bioequivalence certification policy in Chile: An analysis of market authorization data
Source: PLoS One. 2019 May 29;14(5):e0217334. doi: 10.1371/journal.pone.0217334 (PMC6541293; doi:10.1371/journal.pone.0217334)
Supplement: S1 Table — (DOCX) [file pone.0217334.s005.docx]

| **Publication Date** | **Date of Enactment** | **Type** | **No.** | **Description** | **English Translation** |
| --- | --- | --- | --- | --- | --- |
| Sep-96 | Jul-95 | Supreme decree | 1876 | Se determina lista de principios activos contenidos en productos farmacéuticos que requieren demostrar BDP, los criterios para determinarlos, y que las normas y procedimientos para realizar biodisponibilidad los establecerá MINSAL e ISP | A list of active ingredients contained in pharmaceutical products that are required to demonstrate bioavailability, setting out the criteria for determining bioavailability, and the rules and procedures for bioavailability shall be established by MINSAL and ISP |
| Nov-05 | Nov-05 | Exempt Resolution | 727 | Se aprueba la norma que define define los Criterios  para establecer Equivalencia Terapéutica a Productos  Farmacéuticos en Chile | The standard that defines the Criteria to establish Therapeutic Equivalence to Pharmaceutical Products in Chile is approved |
| Nov-05 | Nov-05 | Exempt Resolution | 726 | 1) Lista de 16 PAs contenidos en productos farmacéuticos que deberán hacer estudios "in vivo" para demostrar BDP. 2) Lista de 63 PAs contenidos en productos farmacéuticos que podrán optar a BEX, pudiendo demostrar su BE a través de estudios cinéticos de disolución "in vitro" | 1) List of 16 APIs contained in pharmaceutical products that will have to do "in vivo" studies to demonstrate BDP. 2) List of 63 APIs contained in pharmaceutical products that may be considered under 'biowaiver" [BEX], being able to demonstrate its bioequivalence through kinetic studies of dissolution "in vitro" |
| May-08 | May-08 | Exempt Resolution | 3225 | 1) Se establece fecha de inicio de la exigencia de estudios de BE (2-jul-2008) a productos monodroga que contienen PAs. 2) se establecen productos de referencia. 3) se establece los productos que soliciten registro y que contengan los PAs mencionados, deberán presentar los protocolos de estudios de BDP. 4) los productos con registro vigente, podrán entregar propuesta de cronograma para la realización de estudios de BE respectivos (mes siguiente fecha vigencia de la resolución). y se establecen plazos fatales para presentación de protocolo de estudios de BE. | 1) The start date of the bioequivalence study requirement (July 2, 2008) is for single drug products containing APIs . 2) reference products are established. 3) establishes that any products requesting registration and that contain the APIs mentioned, must present the protocols of studies of bioavailability. 4) any products with current registration, may voluntarily submit a proposal for a schedule for carrying out studies of BE after May 2008 as well as deadlines for submission of BE study protocols. |
| Jan-09 | Dec-08 | Exempt Resolution | 934 | Modifica Resolución 726. 1) Se agregan 36 PAs a la lista de productos farmacéuticos que deberán hacer estudios "in vivo" para demostrar biodisponibilidad. (estos 36 PAs corresponden a aquellos numerados entre el 28 y el 63 de la lista de 63 PAs contenidos en productos farmacéuticos que podrán optar a BEX). 2) los titulares de estos 36 PAs podrán optar a BEX presentando solicitud y antecedentes que fundamenten a ISP (también aplica para los PAs de RES EX 726). 3) ISP determinará referentes y cronograma de trabajo | Modifies Resolution 726. 1) 36 APIs are added to the list of pharmaceutical products that will have to do "in vivo" studies to demonstrate bioavailability. These 36 APIs correspond to those numbered between 28 and 63 of the list of 63 APIs contained in pharmaceutical products that may opt for 'biowaiver'. 2) the holders of these 36 APIs will be able to apply to have biowaiver by submitting an application and background that are based on ISP (also applies to the APIs of RES EX 726). 3) ISP will determine referents and work schedule |

| Dec-09 | Nov-09 | Exempt Resolution | 5555 | Se modifican las resoluciones 728/09 y 2920/09, relativas a exigencias de estudios de BE a productos farmacéuticos monodroga. Suspéndase la exigencia de estudios de bioequivalencia a los siguientes productos monodroga: a) Aquellos que contienen el principio activo Diclofenaco Sódico en comprimidos con recubrimiento entérico en potencias de 25 mg y 50 mg. b) Aquellos que contienen el principio activo Diclofenaco Potásico en comprimidos con recubrimiento entérico en potencias de 12,5 mg, 25 mg y 50 mg. c) Aquellos que contienen el principio activo Didanosina en cápsulas con gránulos con recubrimiento entérico en potencias de 125 mg, 200 mg, 250 mg y 400 mg. | Resolutions 728/09 and 2920/09 relating to the requirements of BE studies for single drug pharmaceutical products are modified. This suspends the BE study requirement with regard to the following single drug products:     A) Those containing the active ingredient Diclofenac Sodium in enteric coated tablets in potencies of 25 mg and 50 mg.     B) Those containing the active ingredient Diclofenac Potassium in enteric coated tablets in potencies of 12.5 mg, 25 mg and 50 mg.     C) Those containing the active ingredient Didanosine in capsules with enteric coated granules in 125 mg, 200 mg, 250 mg y 400 mg. |
| --- | --- | --- | --- | --- | --- |
| Jan-10 | Dec-09 | Exempt Resolution | 5937 | 1) se establece el listado de productos de **referencia** para 36 PAs de la **Resolución 934/08.** 2) los productos **similares** que contengan PAS incluidos en este listado se irán incorporando paulatinamente al régimen de exigencia de estudios de bioequivalencia, al establecer los cronogramas de los mismos, mediante la publicación en el Diario Oficial de las resoluciones respectivas. | 1) establishes the list of reference products for 36 APIs of Resolution 934/08. 2) similar products containing APIs included in this list will gradually be incorporated into the requirement of bioequivalence studies, in establishing the schedules of these, through the publication in the Official Gazette of the respective resolutions. |
| Feb-11 | Jan-11 | Exempt Resolution | 244 | 1) Se establece fecha de inicio de la exigencia de estudios de BE (16-feb-2011) a productos monodroga sólidas orales de **liberación convencional** que contienen los PAs en la(s) dosis señaladas y los referentes. 2) A partir de 16-feb-2011 la **presentación de estudios** de BE y la demostración de equivalencia terapéutica es requisito para el registro de productos que contengan los PAs en la(s) dosis señaladas. 3) los titulares de los productos similares vigentes deberán presentar los estudios en plazo máximo de 12 meses a partir del 16-feb-2011. 4) De no hacerlo se exponen a la cancelación de su registro sanitario. | The start date of the BE study requirement (16-Feb-2011) is established for conventional solid oral single drug products of conventional (i.e., not sustained release) release containing APIs at the indicated doses. 2) As of Feb 16, 2011 the presentation of BE studies and the demonstration of therapeutic equivalence is a requirement for the registration of products containing the APIs at the indicated dose (s). 3) holders of similar products in force must submit the studies within a maximum period of 12 months from Feb 16, 2011. 4) Failure to do so will expose you to cancellation of your health record. |
| Jun-11 | Jan-10 | Supreme decree | 3 | Se aprueba REGLAMENTO DEL SISTEMA NACIONAL DE CONTROL DE LOS PRODUCTOS FARMACÉUTICOS DE USO HUMANO. PÁRRAFO SEGUNDO: DE LA EQUIVALENCIA TERAPÉUTICA.  ARTÍCULO 221º.- El Ministerio de Salud, mediante decreto, aprobará la norma técnica que determine los productos que requieren demostrar su equivalencia terapéutica, estableciendo las listas de los principios activos y de los productos farmacéuticos que servirán de referencia de los mismos, cuando corresponda; lo que podrá hacer a proposición del Instituto.  No requerirán demostrar bioequivalencia las especialidades farmacéuticas contempladas en las letras b), c), d) y e), del artículo 10º del presente reglamento, ni tampoco los preparados farmacéuticos.   Asimismo, por decreto del Ministerio se aprobará la norma técnica que establezca los criterios técnicos necesarios para determinar los productos farmacéuticos que requieran demostrar equivalencia terapéutica.  Las normas y demás procedimientos para la realización de los estudios de biodisponibilidad así como los estudios de equivalencia terapéutica, en los casos que correspondan, serán establecidos por decreto del Ministerio, lo que podrá hacer a proposición del Instituto. | REGULATION OF THE NATIONAL SYSTEM OF CONTROL OF PHARMACEUTICAL PRODUCTS FOR HUMAN USE. SECOND PARAGRAPH: OF THE THERAPEUTIC EQUIVALENCE.      ARTICLE 221.- The Ministry of Health, by decree, shall approve the technical standard that determines the products that need to demonstrate their therapeutic equivalence, establishing the lists of the active principles and of the pharmaceutical products that will serve as reference of the same, when appropriate; Which can be done at the request of the Institute.  The pharmaceutical specialties referred to in Article 10 (b), (c), (d) and (e) of this Regulation shall not be required to demonstrate bioequivalence.      Likewise, by decree of the Ministry, the technical standard that establishes the technical criteria necessary to determine the pharmaceutical products that need to demonstrate therapeutic equivalence will be approved.      The norms and other procedures for the realization of the bioavailability studies as well as the studies of therapeutic equivalence, in the corresponding cases, will be established by decree of the Ministry, which can be done at the request of the Institute. |
| Jun-12 | Jun-12 | Exempt Decree | 500 | APRUEBA NORMA TÉCNICA N° 0136, NOMINADA "NORMA QUE DETERMINA LOS PRINCIPIOS ACTIVOS CONTENIDOS EN PRODUCTOS FARMACÉUTICOS QUE DEBEN DEMOSTRAR SU EQUIVALENCIA TERAPÉUTICA Y LISTA DE PRODUCTOS FARMACÉUTICOS QUE SIRVEN DE REFERENCIA DE LOS MISMOS" 1) Se ratifica y se aprueba lo contenido en **Resolución Exenta 726/11,** relativas a listas de **PAs.** 2) Se aprueban las listas de productos de referencia en las resoluciones exentas **244/11, 728/09, 2920/09, 3225/08 y 5555/09.** 3) Anexo con lista de PAs y productos de referencia. 4) Se podrá demostrar Equivalencia Terapéutica mediante estudios "in vitro", previa solicitud al ISP. [Lo que sigue se estableció en Decreto Exento 904/12] Se otorga plazo de un mes desde la publicacion del presente decreto (6-jun-2012) para presentar estudios de Equivalencia Terapéutica. | **APPROVES TECHNICAL STANDARD N ° 0136,** THAT DETERMINES THE ACTIVE INGREDIENTS CONTAINED IN PHARMACEUTICAL PRODUCTS THAT MUST DEMONSTRATE THEIR THERAPEUTIC EQUIVALENCE AND LIST OF PHARMACEUTICAL PRODUCTS CONTAINING THESE APIs 1) ratifies and approves the content in Exempt Resolution 726/11 concerning lists of APIs . 2) The lists of reference products are approved in exempted resolutions 244/11, 728/09, 2920/09, 3225/08 and 5555/09. 3) Annex with list of APIs and reference products. 4) Therapeutic equivalence may be demonstrated by in vitro studies, upon request to the ISP. [What follows was established in Decree Exempt 904/12] It is granted within one month from the publication of this decree (June 6, 2012) to present studies of TEQ. |

| Sep-12 | Sep-12 | Exempt Decree | 773 | Se modifica el Decreto Exento 500/12. 1) Se incorpora a la "Lista de Productos de Referencia para los Estudios de Equivalencia Terapéutica" a Eutirox (en varias potencias/concentraciones) de Merck S.A. 2) Texto actualizado de la norma en minsal.cl 3) Las disposiciones de este decreto regirán desde la publicación del mismo en el Diario Oficial. | Modified Exempt Decree 500/12. 1) It is incorporated into the "Reference Product List for Therapeutic Equivalence Studies" for levothyroxine sodium (at various strengths / concentrations) of Merck S.A. 2) Current text of the standard in minsal.cl 3) The provisions of this decree will be in force since the publication of the decree in the Official Gazette. |
| --- | --- | --- | --- | --- | --- |
| Oct-12 | Oct-12 | Exempt Decree | 864 | Se modifica el Decreto Exento 500/12. 1) Se incorporan referentes a la "Lista de Productos de Referencia para los Estudios de Equivalencia Terapéutica". Hay productos designado como referente para estudios de Equivalencia Terapéutica, por la autoridad reguladora de la Agencia Nacional de Vigilancia Sanitaria del Brasil. 2) Texto actualizado de la norma en minsal.cl 3) Las disposiciones de este decreto regirán desde la publicación del mismo en el Diario Oficial. 4) Se otorga plazo de un año para cumplir con la exigencia de presentación de estudio de EQT (24-oct-2013) | Modified Exempt Decree 500/12. 1) They are incorporated referring to the "Reference Products List for Therapeutic Equivalence Studies". There are products designated as reference for studies of Therapeutic Equivalence, by the regulatory authority of the National Agency of Sanitary Vigilance of Brazil. 2) Current text of the standard in minsal.cl 3) The provisions of this decree will be in force since the publication of the decree in the Official Gazette. 4) A one-year period is allowed to comply with the requirement to present a TEQ study (meanig that deadline is October 24, 2013) |
| Nov-12 | Nov-12 | Exempt Decree | 904 |  | What follows was established in Decree Exempt 904/12] It is granted within one month from the publication of this decree (June 6, 2012) to present studies of Therapeuctical Equivalence |
| Dec-12 | Dec-12 | Exempt Decree | 981 | Se modifica el Decreto Exento 500/12. 1) se incorporan 98 PAs a la "Lista de Principios Activos contenidos en Productos Farmacéuticos que deberán realizar Estudios Comparativos de Biodisponibilidad In Vivo para demostrar Equivalencia Terapéutica". Se los clasifica entre tres grupos: A,B y C. También se incorporan referentes a la "Lista de Productos de Referencia para los Estudios de Equivalencia Terapéutica". 2) Texto actualizado de la norma en minsal.cl 3) Las disposiciones de este decreto regirán desde la publicación del mismo en el Diario Oficial. 4) se establecen las fechas límites para cumplir con la exigencia de presentar estudios de EQT. Hasta 31-jul-2013 para el grupo A, 31-dic-2013 para el grupo B, y 31-dic-2016 para el grupo C. Esta última fecha también aplica para Levonorgestrel y Nifedipino, cuyos referentes están en este decreto. | Modified Exempt Decree 500/12. 1) 98 APIs are added to the "List of Active Principles contained in Pharmaceutical Products that should perform Comparative In Vivo Bioavailability Studies to demonstrate Therapeutic Equivalence".. They are also incorporated referring to the "Reference Products List for Therapeutic Equivalence Studies". 2) Current text of the standard in minsal.cl 3) The provisions of this decree will be in force since the publication of the decree in the Official Gazette. 4) deadlines are set to meet the requirement to submit studies of therapeutic equivalence. 31-Jul-2013 for group A, 31-Dec-2013 for group B, and 31-Dec-2016 for group C. This last date also applies for Levonorgestrel and Nifedipino, whose referents are in this decree |
| Jun-13 | Jun-13 | Exempt Decree | 633 |  | Modified Exempt Decree 500/12. 1) The conditions and terms granted by exempt decrees 904/12 and 981/12 are modified. It is now July 31, 2014, date to present to the Institute of Public Health the study protocol of In Vivo Therapeutic Equivalence. December 31, 2013, date to present to the Institute of Public Health the Finished Study of Therapeutic Equivalence, In Vivo or In Vitro, as appropriate. 2) 39 APIs will be eligible for biowaiver (the list is in the decree) |
| Oct-13 | Oct-13 | Exempt Decree | 1067 |  | Exempt Decree 500/12 is modified. 1) Modifies the term granted by exempt decree 864/12, from October 24, 2013, to April 30, 2014, to Present to the Institute of Public Health the Finished Study of Therapeutic Equivalence, In Vivo or In Vitro, as appropriate. 2) Current text of the standard in minsal.cl |
| Dec-14 | Dec-14 | Exempt Decree | 1299 |  | MODIFY DECREE 981/12. 1) Amend article 4 of exempt decree 981/12 to replace the demonstration period of therapeutic equivalence established for the pharmaceutical products included in Group C of the same article by December 31, 2015. 2) Obligation indicated in the previous article shall be deemed to have been fulfilled once the holder of the registration submits to the Chilean Public Health Institute the Final Report of the Bioequivalence or Biowaiver Study. |
